# Human Papillomavirus Infection and Oropharyngeal and Gastrointestinal Cancers: A Causal Relationship?

**DOI:** 10.3390/diseases10040094

**Published:** 2022-10-20

**Authors:** Zeynep Deniz, Suleyman Uraz, Ryan Holem, Resat Ozaras, Veysel Tahan

**Affiliations:** 1 School of Medicine, Acıbadem Mehmet Ali Aydınlar University, Istanbul 34755, Turkey; 2 Department of Gastroenterology, School of Medicine, Demiroglu Bilim University, Istanbul 34394, Turkey; 3 Department of Gastroenterology, University of Missouri School of Medicine, Columbia, MO 65212, USA; 4 Infectious Diseases Department, Medilife Hospital, Yakuplu Mah, Hurriyet Bulvari, No: 5, TR-34524 Beylikduzu, Istanbul 34523, Turkey

**Keywords:** human papillomavirus, oropharyngeal cancer, esophageal cancer, gastric cancer, colorectal cancer, anal cancer

## Abstract

The human papillomavirus (HPV) is one of the most common sexually transmitted infections worldwide. The risk of being infected at least once in a lifetime among both men and women is estimated to be 50%. Although the majority of HPV infections are asymptomatic and improve within 2 years, approximately 10% of individuals develop a persistent infection and have an increased risk of developing carcinomas. The association of HPV and genital cancer is well established. However, there is evidence that HPV may also be associated with other cancers, including those of the gastrointestinal system. The aim of this review is to organize the current evidence of associations between HPV infections and oropharyngeal and gastrointestinal cancers, including the following: oropharyngeal, esophageal, gastric, colorectal, and anal cancers. A comprehensive review of the most up-to-date medical literature concluded that an HPV infection might have a role in the oncogenesis of gastrointestinal tract cancers. HPV may have a causal relationship with oropharyngeal and esophageal squamous cell cancers. However, the association between HPV and gastric and colorectal cancers is weaker. The development of cancer in the oropharyngeal and gastrointestinal tract is usually multifactorial, with HPV having a role in at least a subset of these cancers. HPV infections pose a big challenge due to their burden of infection and their oncogenic potential.

## 1. Description of the Human Papillomavirus (HPV)

The human papillomavirus (HPV) is a small, non-enveloped, double-stranded DNA virus belonging to the papillomavirus family. HPV is one of the most common sexually transmitted infections worldwide. The risk of being infected at least once in a lifetime among both men and women is estimated to be 50% [1]. The transmission of HPV is through personal contact. Anogenital warts are commonly sexually transmitted. The age of onset of an HPV infection is similar to that of other sexually transmitted diseases (STDs). Additionally, individuals with HPV have higher frequencies of STDs, and having a large number of sexual partners is associated with a greater risk of condylomata acuminata [2]. However, minor trauma at the site of infection may also have a role since HPV incidence is higher among meat handlers, such as butchers. Young children may also acquire the virus from hand contact with non-genital lesions. Observations suggest that the disease may be transmitted through vaginal delivery and in utero via ascending infection from the mother’s genital tract [3].

HPV has an established oncogenic potential and is a major cause of infection-related cancers worldwide. HPV is diagnosed in more than 90% of cervical cancers and is currently the most common pathogen responsible for cancers in women [4]. Besides cervical cancers, HPV infections have been reported to potentially be associated with 90–93% of anal cancers, 12–63% of oropharyngeal cancers, 36–40% of penile cancers, 40–64% of vaginal cancers, and 40–51% of vulvar cancers [5]. The oncogenic potential of HPV varies according to genotype; the most common genotypes of HPV are 16 and 18, which are among the high-risk genotypes (Table 1). Both HPV type 16 and HPV type 18 are vaccine-preventable [1].

HPV infections, especially with high-risk genotypes, have been recently associated with gastrointestinal cancers. Although direct evidence is lacking and the results of the studies are inconsistent, serologic and histologic studies suggest a possible association.

## 2. HPV and Oncogenesis

Oncogenic HPVs are sexually transmitted, and the infection (typically cervical) can result in mild cytological abnormalities [8]. For the majority of people (around 90%), it improves within 2 years. In a small group of patients, it causes long-term persistent infections, leading to cervical intraepithelial neoplasia (CIN). The oncogenic progression from CIN1 to CIN3 may lead to invasive cervical cancer. It is estimated that one third of CIN3 can progress to cancer within 10 to 20 years [8]. 

The HPV proteins E6 and E7 have a critical role in HPV-induced cellular immortalization, transformation, and carcinogenesis [9]. Both E6 and E7 express their oncogenic activity primarily through protein–protein interactions with tumor suppressor proteins. The tumor suppressor p53 has a role in preventing the development of cancer. In healthy cells, p53 levels are kept low by degradation via the ubiquitin ligase Mdm2 [10]. Alterations of p53 are common in cancer. The E6 oncoprotein of HPV forms a ternary complex with the cellular E3 ubiquitin ligase E6AP and p53, leading to ubiquitin-mediated degradation of p53 and a subsequent decrease in its cellular levels [11,12]. A common polymorphism occurring in the p53 amino acid sequence results in the presence of either a proline or an arginine at position 72 and increases the susceptibility of p53 to E6-mediated degradation [11,13]. The E7 protein binds to the retinoblastoma protein 1 (RB1) and provides its inactivation, leading to cell-cycle progression through the activation of E2F-driven transcription [14]. 

During the persistent infection, the virus is presumably not detected by the immune system [15]. Since the viral activity is mainly in the upper cell layers, the virus may escape the immune responses. In addition, HPV interferes with the innate immune responses and has the ability to delay the adaptive immune responses [15]. An HPV infection follows a latent course, and thereafter it may reactivate. Re-infection is possible even with the same HPV type, especially in women who previously did not seroconvert, suggesting the protective role of humoral immunity [8]. The role of the immune system in controlling HPV infections and subsequent cancer development is evident in individuals with immunosuppression. Patients with Fanconi’s anemia have DNA repair defects and are more susceptible to HPV-related cancers [16]. 

Chronic inflammation of the digestive system by an HPV infection seems to cause long-term complications. However, an infection with a non-cancerogenic HPV type may provide protection from the cancerogenic types through cross-reactivity, epitope spreading, and de novo immune stimulation [17]. 

## 3. Human Papillomavirus and Gastrointestinal Cancers

There is a clear association between HPV and genital cancers. Additionally, there is evidence that HPV may have a role in the oncogenesis of oropharyngeal and anal cancers. Increasing epidemiological information suggests that even other gastrointestinal cancers may have an association with HPV. The medical literature is complicated by the sample size, the detection method for HPV, and the definition of a control group among studies. The HPV detection methods, the specimens used, and the advantages and disadvantages of the methods are given in Table 2. 

### 3.1. HPV and Oropharyngeal Cancer

Oropharyngeal cancer (OPC) includes tumors that arise from the oral cavity, oropharynx, larynx, hypopharynx, and sinonasal tract. The main risk factors are male gender, prior tobacco use, and alcohol use [21]. About 90% of head and neck cancers are squamous cell carcinomas. HPV is suggested to have a role in oropharyngeal cancers, particularly in squamous cell carcinomas of the region. 

While the incidence of head and neck cancers has decreased in the last few decades, the incidence of OPCs has increased and is largely attributable to the increase in HPV infections [22]. The prevalence of HPV-positive OPCs has increased from approximately 47% in 2000 to approximately 74% in 2012 in Canada [23]. It has also increased by 225% between 1988 and 2004 and now comprises up to 90% of all new cases of OPCs in the United States [24]. Current evidence shows a causal relationship between oropharyngeal squamous cell carcinoma (SCC) and HPV. The high-risk HPV types 16 and 18 appear to be the cause of the rising rate of SCCs [22,23,24]. The patients’ characteristics also have changed; the patients are often younger and healthier with a high socioeconomic status and with minimal to no smoking history, in contrast to the traditional older patient with a long history of tobacco and alcohol use [25]. Compared to HPV-negative OPCs, after treatment, HPV-positive OPCs are associated with a more favorable prognosis. 

HPV-positive OPCs have a male predominance; their incidence in men is three to five times higher than in women worldwide [26]. More than 90% of HPV-positive OPCs are caused by the high-risk HPV genotype 16 [26].

### 3.2. HPV and Esophagus Cancer

Esophageal carcinoma (EC) is one of the most aggressive malignant cancers of the gastrointestinal tract. Multiple risk factors are defined for the carcinogenesis, including alcohol and tobacco use, nutritional deficiencies, and infectious agents [27]. HPV infections have been recently reported as another possible risk factor for the development of ECs. 

There are conflicting results in the studies investigating the role of HPV. The studies mainly compared tissues with cancer to surrounding normal tissues and studied HPV by PCR or immunochemistry [28,29,30,31,32,33,34,35,36,37,38,39,40,41,42,43,44,45,46,47,48,49,50,51,52,53,54,55,56,57,58,59,60,61,62,63,64,65,66,67,68,69,70,71] (Table 3). Among the studies, 28 out of 44 (64%) reported that HPV positivity is significantly higher in esophageal cancer patients, including the studies enrolling the highest number of patients [33,55,60,67,69]. We included studies with all histologic types. Studies that focused on esophageal squamous cell carcinomas (ESCCs) suggested a stronger association as follows: an analysis of eight meta-analyses studying the role of HPV reported that the meta-analyses included between 1223 and 13,832 patients with ESCCs and the overall percentage of HPV-positive ESCC cases ranged from 11.7% to 38.9%. The analysis reported that geographic location accounts for a majority of the variations in HPV prevalence; high-incidence regions, including Asia, reported significantly higher HPV–ESCC infection rates compared to low-incidence regions, such as Europe, North America, and Oceania [72]. 

The meta-analysis and the systematic review included 33 randomized studies with 6051 EC patients [73]. The HPV infection rate in the EC group was 46.5% (1131/2430), while it was 26.2% (977/3621) in the control group (OR = 1.62; 95% CI 1.33–1.98). This meta-analysis suggests that HPV infections and the incidence of ECs are closely associated.

### 3.3. HPV and Gastric Cancer

Gastric cancer (GC) is the sixth most common cancer in both sexes worldwide [74]. It is more prevalent in the male population in developing countries, mainly in East Asia, South America, and Eastern Europe. The etiological factors include the following: improper eating habits, a diet rich in cured and smoked meat products with low antioxidant content, tobacco smoking and alcohol consumption, and chronic *Helicobacter pylori* or Epstein–Barr virus (EBV) infections [75,76]. The role of HPV in the oncogenesis of gastric cancer has been investigated in a few studies. 

A meta-analysis published in 2016 investigated the HPV and gastric carcinoma association [77]. It included 30 studies with 1917 patients with GC and 576 controls. It found the pooled HPV prevalence to be 28.0%. Based on 15 case control studies, the pooled odds ratio was found to be 7.388. The meta-analysis found that the HPV prevalence was significantly higher in patients from China than in those from non-Chinese regions (31% vs. 9%, *p* < 0.001). The pooled prevalence of HPV 18 was found to be 7% and HPV 16 was found to be 21% in GC tissues. HPV 16 was detected as being three times more prevalent than HPV 18.

Another recent meta-analysis studied the role of HPV in GC [78]. It included 14 studies investigating the prevalence of HPV in 901 gastric carcinoma patients and 1205 controls. The pooled HPV prevalence in gastric carcinoma was found to be 23.6%. An association between an HPV infection and a risk of GC was observed (odds ratio = 1.53). In this meta-analysis, noncancerous, healthy, and benign gastric diseases were used as controls, and the prevalence of HPV in patients with GC was significantly higher than that in the paired corresponding normal tissue controls. The association between an HPV-16 infection and a risk of gastric cancer was statistically significant (OR = 2.42). 

HPV type 16 infection seemed to increase the risk of GC. However, since GC oncogenesis is multifactorial and it is not clear whether the effect of HPV is due to its oncogenic potential or whether it is the consequence of chronic inflammation; a causal relationship remains to be established. It is not clear whether the prognosis for HPV-positive GC differs from the HPV-negative one.

### 3.4. HPV and Colorectal Cancer

Colorectal cancer (CRC) is the second most common cancer in women and the third most common one in men [79]. The risk factors are reported as heredity (genetic factors), lifestyle factors, such as high consumption of red meat, or tobacco smoking [80]. HPV can infect the colorectal region via an ascending infection from anogenital sites or through hematogenous or lymphatic spread. HPV has been suggested as a potential risk factor for the development of CRCs. Many studies investigated the presence of HPV in colorectal cancer tissues and compared them to normal and surrounding tissues (Table 4) [81,82,83,84,85,86,87,88,89,90,91,92,93,94,95,96,97,98,99,100,101,102,103,104,105,106,107,108,109,110,111]. In the majority of cases, the prevalence of HPV in cancer tissue has been higher than that in the control, suggesting a role for HPV in the oncogenesis of CRC. 

The correlation between HPV and CRC was analyzed using the Hill criteria [112]. The analysis revealed some evidence for analogy, biological plausibility, and strength of association but only weak evidence for consistency, specificity, and coherence. Two meta-analyses suggested a significant increase in the risk of CRC development with the presence of HPV [113,114]. However, the sample sizes were small and the HPV detection methods varied among studies. 

Among 32 controlled studies, 17 (53%) found a higher prevalence of HPV in colorectal cancer patients. Studies involving the highest numbers of patients were among those finding no significant differences [84,98,105]. The current evidence shows an increased risk, and further information is needed to establish a causal relationship. There is no information on the clinical outcomes of HPV-associated colorectal tumors. 

### 3.5. HPV and Anal Cancer

The majority of anal cancers (89–100%) are induced by long-term HPV infections [115]. Anal cancer comprises 4% of all malignancies of the lower alimentary tract. Its incidence is increasing in high-income countries, and its incidence is higher in women than in men [113]. The risk groups for anal cancer have been identified as men who have sex with men, transplant recipients, people living with HIV or AIDS infections, and women with a history of HPV-induced cervical, vaginal, or vulvar cancer [115]. 

There are an estimated 27,000 new cases of anal cancer every year worldwide, with the ratio of female to male cases being as high as 5:1 [116]. White women have higher rates of anal squamous cell carcinomas compared to black women. On the other hand, black men have a significantly higher incidence of anal cancer than white men [117]. 

HPV infections are not necessarily transmitted directly to the anus; they can spread from one area to another. They can extend from the genitals to other organs. They can be transmitted not only through sexual intercourse (vaginal, anal, or oral), but also via hand-to-genital contact. Many HPV-infected individuals may not have genital warts or other signs of infection [116]. During an active HPV infection, patients may present with common warts, plantar warts, flat warts, anal dysplasia, or oropharyngeal lesions. In particular, there can be genital warts with various appearances, including flat, raised, single, or multiple. 

There may be no symptoms in 20% of individuals, but the majority of individuals can present symptoms, including fissures, hemorrhoids, dermatitis of the anal region, pain in the anal region, loss of bowel control, and anorectal fistulas with discharge [118]. A growing mass in the anal canal may appear later, or the patient may present with inguinal lymphadenopathy due to metastasis. 

There appears to be a higher prevalence of HPV in patients with oropharyngeal and gastrointestinal cancers, suggesting a role for HPV in the oncogenesis of gastrointestinal tract cancers. In parallel to the urogenital cancers, the prevalent HPV types are 16 and 18 in oropharyngeal and gastrointestinal cancers (Table 5). 

In squamous oropharyngeal and squamous esophageal cell cancers, the current evidence points to a causal relationship. In gastric and colorectal cancers, the association seems weaker. The development of cancer in the oropharyngeal and gastrointestinal tract is usually multifactorial; HPV may have a role in at least a subset of these cancers. HPV infections pose a big challenge due to their burden of infection and oncogenic potential. The HPV strains that might be involved in the oncogenesis of oropharyngeal and gastrointestinal tract cancers are vaccine-preventable. HPV vaccination is key not only to the prevention of urogenital cancers but also, at least in part, of oropharyngeal and gastrointestinal tract cancers, since the study of the bivalent HPV 16/18 vaccine in women showed that cervical, anal, and oral infections could be prevented [119]. The screening of clinical samples of the oropharyngeal and gastrointestinal systems for HPV can provide more information about the relationship and provide the detection of HPV-positive individuals. The relatively higher prevalence of HPV among patients with oropharyngeal and gastrointestinal cancers should be further investigated by basic and clinical studies to clarify any causal relationship. 

## 4. Conclusions

Persistent HPV infections induce the development of cancer. While a clear association has been established with urogenital cancers, the current evidence also points to a causal relationship with squamous oropharyngeal and squamous esophageal cell cancers. For gastric and colorectal cancers, HPV may have a role, although the association is weaker. 

The most prevalent HPV types, 16 and 18, in urogenital cancers are also prevalent in oropharyngeal and gastrointestinal cancers. Since these types are vaccine-preventable, HPV vaccination is the optimal strategy to prevent HPV-related cancers, including urogenital cancers and at least a certain part of oropharyngeal and gastrointestinal tract cancers. 

The relatively higher prevalence of HPV among patients with oropharyngeal and gastrointestinal cancers should be further studied. The screening of clinical samples of the oropharyngeal and gastrointestinal systems for HPV can provide the greatest number of patients to obtain more conclusive information.

## Figures and Tables

**Table 1:** Human papillomavirus genotypes and their oncogenic risks [6,7].

	Genotypes	Characteristics
High risk	16, 18, 31, 33, 35, 39, 45, 51, 52, 56, 58, 59, 68, 73, 82	They are associated with cancers.
Low risk	6, 11, 40, 42, 43, 44, 53, 54, 61, 72, 81	They cause benign lesions affecting the anogenital areas, such as genital warts (condylomata), low-grade squamous intraepithelial lesions of the cervix, and laryngeal papillomas.

**Table 2:** Human papillomavirus detection methods [18,19,20].

	Specimen	Advantage	Disadvantage
Detection of HPV Proteins			
Immunohistochemistry	Fresh-frozen samples, FFPE	High experience, commonly used, high sensitivity	Low specificity, time consuming, technically cumbersome
Western blot	Fresh-frozen samples	Moderate to high specificity	Low sensitivity, time consuming, technically cumbersome
Detection of HPV genomes			
Southern blot	Fresh-frozen samples	Moderate to high sensitivity and specificity	Not easily performed to FFPE tissues
In situ hybridization	FFPE, fresh samples	Moderate sensitivity and specificity	Requires DNA and tissue preservation
Signal amplification (hybrid capture)	Fresh-frozen samples, FFPE	High sensitivity and specificity, provides viral load information	No typing provided
PCR	Fresh-frozen samples, any bodily fluid	Very high sensitivity, low specificity	Does not provide quantitative measurement of viral load
Real-time PCR	Fresh-frozen samples, FFPE tissues, any bodily fluid	Very high sensitivity and specificity	Labor-intensive
Detection of antibodies against HPV	Serum	Easy to perform	Sensitivity and specificity are low

PCR, polymerase chain reaction; FFPE, formalin-fixed and paraffin-embedded.

**Table 3:** Summary of the studies on the human papillomavirus in esophageal cancer.

Reference	Country	Year	HPV (+) in Cancer Patients	HPV (+) in Control Group	*p* *	HPV Detection Method	HPV Types Studied
Astori, G., et al. [28]	Italy	2001	7/17	4/16	NS	PCR	16
Acevedo, N.E., et al. [29]	Mexico	2004	15/17	4/23	<0.00001	PCR	11,16
Bahnassy, A.A., et al. [30]	Egypt	2005	27/50	12/50	<0.05	PCR	11,16,18
Benamouzig, R., et al. [31]	France	1992	5/12	1/24	<0.05	ISH	6,11,16,18,31,33
Cao, B.W., et al. [32]	China	2005	207/265	203/357	<0.00001	PCR	16,18
Chen, J., et al. [33]	China	2004	14/30	7/60	<0.001	PCR	16
Chen, W.G., et al. [34]	China	2014	44/66	8/66	<0.00001	Gene chip technology	16,18
Cui, X., et al. [35]	Kazakhstan	2014	58/183	8/89	<0.0001	PCR	6,11,16,18,35,43,52
da Costa, A.M., et al. [36]	Brazil	2018	12/87	12/87	NS	PCR	16
Dąbrowski, A., et al. [37]	Poland	2012	28/56	4/35	NS	PCR	16,18
Dong, H.C., et al. [38]	Kazakhstan	2015	46/89	14/49	<0.00001	PCR	6,16,33,39,51,82
Farhadi, M., et al. [39]	Iran	2005	14/38	5/38	<0.05	PCR	16,18
Georgantis, G., et al. [40]	Greece	2015	2/19	0/30	NS	PCR	11,31
Gessner, A.L., et al. [41]	Malawi	2018	6/40	0/12	NS	RT-PCR and ISH	16,18,31,45
Han, C., et al. [42]	China	1996	22/90	6/121	<0.0001	ELISA	16
He, Z., et al. [43]	China	2014	366/1435	213/2071	<0.00001	ELISA	16
Jiang, H.Y., et al. [44]	China	2005	48/65	11/65	<0.00001	IHC	16,18
Kamangar, F., et al. [45]	China	2006	33/99	106/381	NS	ELISA	16,18,73
Kawaguchi, H., et al. [46]	Japan, China	2000	17/75	17/75	NS	PCR	16
Kayamba, V., et al. [47]	Zambia	2015	2/44	1/48	NS	PCR	-
Khurshid, A., et al. [48]	Japan	1998	17/27	3/12	<0.05	PCR	16,18,33
Kuang, Z.S., et al. [49]	China	2000	23/56	4/56	<0.0001	PCR	16,18
Lagergren, J., et al. [50]	Sweden	1999	20/193	61/302	<0.05	ELISA	16,18
Li, Y., et al. [51]	China	1991	12/24	9/24	NS	ISH	16
Liu, H.Y., et al. [52]	China	2014	11/30	60/78	<0.0001	PCR	16
Liu, J., et al. [53]	China	2000	44/60	23/56	0.001	IHC	16,18
Liyanage, S.S., et al. [54]	Australia	2014	1/99	0/100	NS	PCR-ELISA	16
Lu, L.C., et al. [55]	China	1999	15/55	43/55	<0.00001	ISH	18
Lu, X.M., et al. [56]	China	2004	55/104	41/104	0.051 (NS)	PCR	16
Lyronis, I.D., et al. [57]	Greece	2008	14/22	3/14	<0.05	PCR	16,18
Parameshwaran, K., et al. [58]	Australia	2019	6/41	1/49	<0.05	PCR	16,18
Rajendra, S., et al. [59]	Australia	2020	3/26	5/328	<0.001	ELISA	6,11,16,18,31,33,45,52,58
Ren, Z.H.P., et al. [60]	China	1996	35/52	2/30	<0.00001	IHC	16,18
Sadeghian, Z., et al. [61]	Iran	2022	20/70	0/70	<0.00001	PCR	16,18
Shen, Z.Y., et al. [62]	China	2002	115/176	105/176	NS	PCR	6,11,16,18
Tornesello, M.L., et al. [63]	Italy	2009	12/56	8/27	NS	PCR	6,8,15,16,20,25
Wang, X.J., et al. [64]	China	1998	20/40	36/58	NS	ISH	16
Wong, M.Y.W., et al. [65]	Australia	2018	18/36	6/55	<0.0001	PCR	6,16,18
Xu, C.L., et al. [66]	China	2004	16/18	126/183	NS	IHC	16
Xu, W.G., et al. [67]	China	2003	28/40	10/50	<0.00001	ISH	16
Yahyapour, Y., et al. [68]	Iran	2018	27/100	28/68	0.054(NS)	Real Time PCR	16,18,35,39,45,56
Yang, J., et al. [69]	China	2014	170/313	136/314	<0.01	ELISA	16
Zhou, S.M., et al. [70]	China	2009	26/82	10/80	<0.05	PCR	16
Zhou, X.B., et al. [71]	China	2003	31/48	8/23	<0.05	PCR	16

* Chi-square test. NS, not significant; PCR, polymerase chain reaction; IHC, immunohistochemistry; ISH, in situ hybridization; ELISA, enzyme-linked immunosorbent assay.

**Table 4:** Summary of the studies on the human papillomavirus in colorectal cancer.

Reference	Country	Year	HPV Positivity in Cancer Tissues	HPV Positivity in Control Tissues	*p* *	Detection Method	HPV Types Studied
Aghakhani, A., et al. [81]	Iran	2014	0/70	0/30	NS	PCR	>20 types
Audeau, A., et al. [82]	New Zealand	2002	20/2351	0/20	NS	IHC	6,11,16,18
Bernabe-Dones, R.D., et al. [83]	USA	2016	19/45	1/36	<0.0001	nested PCR	>25 types
Bodaghi, S., et al. [84]	USA	2005	28/55	0/10	<0.01	PCR	>20 types
Boguszaková, L., et al. [85]	Czechia	1998	0/13	0/10	NS	Southern blot hybridization	2,6,16,18
Buyru, N., et al. [86]	Turkey	2006	43/53	17/53	<0.00001	PCR	6,11,16,18,33
Cheng, J.Y., et al. [87]	China	1995	37/70	11/37	<0.05	PCR and southern blot	6,11,16,18,33
Dalla Libera, L.S., et al. [88]	Brazil	2020	13/79	0/75	<0.001	PCR	32 types
Gazzaz, F., et al. [89]	Saudi Arabia	2016	2/60	2/72	NS	Digene procedure	18 types
Gornick, M.C., et al. [90]	USA	2010	0/279	0/30	NS	PCR reverse line blot and the SPF10 INNO-LiPA	39 types
Hafez, F.S., et al. [91]	Egypt	2022	13/59	0/30	<0.01	IHC	-
Kirgan, D., et al. [92]	USA	1990	42/43	25/60	<0.00001	IHC	6,11,16,18,31,33,35
			18/42	5/25	NS	In situ DNA hybridization	
Laskar, R.S., et al. [93]	India	2015	34/93	6/30	NS	PCR	16,18
Lee, Y.M., et al. [94]	Taiwan	2001	16/19	10/19	<0.05	PCR and southern blot	18
Li, Y.X., et al. [95]	China	2015	46/95	29/100	<0.01	HPV Genotyping Chip technology and IHC	23 types
Liu, F., et al. [96]	China	2011	32/96	0/32	<0.0001	PCR	21 types
Mahmoudvand, S., et al. [97]	Iran	2015	2/70	4/140	NS	PCR	16,18
McGregor, B., et al. [98]	USA	1993	13/38	10/45	NS	PCR and southern blot	6,11,16,18,33
Militello, V., et al. [99]	Italy	2009	2/72	0/72	NS	PCR	>20 types
Nagi, K., et al. [100]	Lebanon	2021	60/94	4/13	<0.05	PCR	14 types
Pérez, L.O., et al. [101]	Argentina	2005	40/54	10/30	<0.001	PCR	>20 types
Picanço-Junior, O.M., et al. [102]	Brazil	2014	36/79	5/65	<0.00001	PCR	19 types
Ranjbar, R., et al. [103]	Iran	2014	5/80	1/80	NS	nested PCR	>20 types
Salepci, T., et al. [104]	Turkey	2009	46/56	18/56	<0.0001	PCR and southern blot hybridization	6,11,16,18,33
Snietura, M., et al. [105]	Poland	2012	0/10	0/40	NS	qPCR	14 types
Soto, Y., et al. [106]	Cuba	2016	10/24	5/39	<0.01	qPCR	16,18,31,33,45,52,58
Taherian, H., et al. [107]	Iran	2014	0/50	0/50	NS	PCR	-
Tanzi, E., et al. [108]	Italy	2015	9/57	5/57	NS	Degenerate PCR	16,18,33,58
Vuitton, L., et al. [109]	France	2017	0/210	0/40	NS	qPCR	28 types
Zhang, J., et al. [110]	China	2005	42/82	4/82	<0.00001	PCR	16
Zhu, Q., et al. [111]	China	1999	20/46	0/16	<0.01	PCR	6,11,16,18,33

* Chi-square test. NS, not significant; PCR, polymerase chain reaction; qPCR, quantitative PCR; IHC, immunohistochemistry.

**Table 5:** Prevalent HPV types in urogenital and gastrointestinal cancers [6,7].

	HPV Type
Urogenital cancers	16 (squamous cell carcinomas), 18 (adenocarcinomas), 45,31,33,52,58,35
Gastrointestinal cancers	
Oropharyngeal cancers	16 (>90%)
Esophageal cancers	16,18
Gastric cancers	16,18
Colorectal cancers	16,18,31,33
Anal cancers	16 (80%), 18

## Data Availability

Data sharing not applicable.

## References

[1] Brianti P., De Flammineis E., Mercuri S.R. (2017). Review of HPV-related diseases and cancers. New Microbiol..

[2] Bonnez W., Reichman R.C. (2010). Papillomaviruses. Mandell, Douglas, and Bennett’s Principles and Practice of Infectious Diseases.

[3] Derkay C.S. (2006). Recurrent respiratory papillomatosis. Ann. Otol. Rhinol. Laryngol..

[4] Chaturvedi A.K. (2010). Beyond cervical cancer: Burden of other HPV-related cancers among men and women. J. Adolesc. Health..

[5] de Martel C., Ferlay J., Franceschi S., Vignat J., Bray F., Forman D., Plummer M. (2012). Global burden of cancers attributable to infections in 2008: A review and synthetic analysis. Lancet Oncol..

[6] Haręża D.A., Wilczyński J.R., Paradowska E. (2022). Human papillomaviruses as infectious agents in gynecological cancers. Oncogenic properties of viral proteins. Int. J. Mol. Sci..

[7] Trottier H., Burchell A.N. (2009). Epidemiology of mucosal human papillomavirus infection and associated diseases. Public Health Genom..

[8] Gravitt P.E. (2011). The known unknowns of HPV natural history. J. Clin. Investig..

[9] Venuti A., Paolini F., Nasir L., Corteggio A., Roperto S., Campo M.S., Borzacchiello G. (2011). Papillomavirus E5: The smallest oncoprotein with many functions. Mol. Cancer.

[10] Trave G., Zanier K. (2016). HPV-mediated inactivation of tumor suppressor p53. Cell Cycle.

[11] Huibregtse J.M., Scheffner M., Howley P.M. (1991). A cellular protein mediates association of p53 with the E6 oncoprotein of human papillomavirus types 16 or 18. EMBO J..

[12] Werness B.A., Levine A.J., Howley P.M. (1990). Association of human papillomavirus types 16 and 18 E6 proteins with p53. Science.

[13] Storey A., Thomas M., Kalita A., Harwood C., Gardiol D., Mantovani F., Breuer J., Leigh I.M., Matlashewski G., Banks L. (1998). Role of a p53 polymorphism in the development of human papillomavirus-associated cancer. Nature.

[14] Dyson N., Howley P.M., Münger K., Harlow E. (1989). The human papilloma virus-16 E7 oncoprotein is able to bind to the retinoblastoma gene product. Science.

[15] Stanley M.A. (2012). Epithelial cell responses to infection with human papillomavirus. Clin. Microbiol. Rev..

[16] McBride A.A. (2017). Oncogenic human papillomaviruses. Philos. Trans. R. Soc. B.

[17] Nakagawa M., Greenfield W., Moerman-Herzog A., Coleman H.N. (2015). Cross-reactivity, epitope spreading, and de novo immune stimulation are possible mechanisms of cross-protection of nonvaccine human papillomavirus (HPV) types in recipients of HPV therapeutic vaccines. Clin. Vaccine Immunol..

[18] Villa L.L., Denny L. (2006). Methods for detection of HPV infection and its clinical utility. Int. J. Gynecol. Obstet..

[19] Abreu A.L., Souza R.P., Gimenes F., Consolaro M.E. (2012). A review of methods for detect human Papillomavirus infection. Virol. J..

[20] Bucchi D., Stracci F., Buonora N., Masanotti G. (2016). Human papillomavirus and gastrointestinal cancer: A review. World J. Gastroenterol..

[21] Elrefaey S., Massaro M., Chiocca S., Chiesa F., Ansarin M. (2014). HPV in oropharyngeal cancer: The basics to know in clinical practice. Acta Otorhinolaryngol. Ital..

[22] Guo T., Eisele D.W., Fakhry C. (2016). The potential impact of prophylactic human papillomavirus vaccination on oropharyngeal cancer. Cancer.

[23] Habbous S., Chu K.P., Lau H., Schorr M., Belayneh M., Ha M.N., Murray S., O'Sullivan B., Huang S.H., Snow S. (2017). Human papillomavirus in oropharyngeal cancer in Canada: Analysis of 5 comprehensive cancer centres using multiple imputation. Can. Med. Assoc. J..

[24] Chaturvedi A.K., Engels E.A., Pfeiffer R.M., Hernandez B.Y., Xiao W., Kim E., Jiang B., Goodman M.T., Sibug-Saber M., Cozen W. (2011). Human papillomavirus and rising oropharyngeal cancer incidence in the United States. J. Clin. Oncol..

[25] You E., Henry M., Zeitouni A.G. (2019). Human papillomavirus–Associated oropharyngeal cancer: Review of current evidence and management. Curr. Oncol..

[26] Pan C., Issaeva N., Yarbrough W.G. (2018). HPV-driven oropharyngeal cancer: Current knowledge of molecular biology and mechanisms of carcinogenesis. Cancers Head Neck.

[27] Rubenstein J.H., Shaheen N.J. (2015). Epidemiology, diagnosis, and management of esophageal adenocarcinoma. Gastroenterology.

[28] Astori G., Merluzzi S., Arzese A., Brosolo P., de Pretis G., Maieron R., Pipan C., Botta G.A. (2001). Detection of human papillomavirus DNA and p53 gene mutations in esophageal cancer samples and adjacent normal mucosa. Digestion.

[29] Acevedo-Nuño E., González-Ojeda A., Vázquez-Camacho G., Balderas-Pena L.-M., Moreno-Villa H., Montoya-Fuentes H. (2004). Human papillomavirus DNA and protein in tissue samples of oesophageal cancer, Barrett’s oesophagus and oesophagitis. Anticancer Res..

[30] Bahnassy A.A., Zekri A.-R.N., Abdallah S., El-Shehaby A.M.R., Sherif G.M. (2005). Human papillomavirus infection in Egyptian esophageal carcinoma: Correlation with p53, p21waf, mdm2, C-erbB2 and impact on survival. Pathol. Int..

[31] Benamouzig R., Pigot F., Quiroga G., Validire P., Chaussade S., Catalan F., Couturier D. (1992). Human papillomavirus infection in esophageal squamous-cell carcinoma in western countries. Int. J. Cancer.

[32] Cao B.W., Yu J., He H., Li S., Zhao Y. (2010). Meta analysis on the relationship between HPV infection and esophageal cancer in Chinese population. J. Cap. Med. Univ..

[33] Chen J., Zhou Y., Li J., Wu P. (2004). Effect of HPV16 infection on the EC and the gene expressions of P53 and P16. Chin. J. Lab. Diagn..

[34] Chen W.-G., Yang C.-M., Xu L.-H., Zhang N., Liu X.-Y., Ma Y.-G., Huo X.-L., Han Y.-S., Tian D.-A., Zheng Y. (2014). Gene chip technology used in the detection of HPV infection in esophageal cancer of Kazakh Chinese in Xinjiang Province. J. Huazhong Univ. Sci. Technol..

[35] Cui X., Chen Y., Liu L., Li L., Hu J., Yang L., Liang W., Li F. (2014). Heterozygote of PLCE1 rs2274223 increases susceptibility to human papillomavirus infection in patients with esophageal carcinoma among the Kazakh populations. J. Med. Virol..

[36] da Costa A.M., Fregnani J.H.T.G., Pastrez P.R.A., Mariano V.S., Neto C.S., Guimarães D.P., de Oliveira K.M.G., Neto S.A.Z., Nunes E.M., Ferreira S. (2018). Prevalence of high risk HPV DNA in esophagus is high in Brazil but not related to esophageal squamous cell carcinoma. Histol. Histopathol..

[37] Dabrowski A., Kwaśniewski W., Skoczylas T., Bednarek W., Kuźma D., Goździcka-Józefiak A. (2012). Incidence of human papilloma virus in esophageal squamous cell carcinoma in patients from the Lublin region. World J. Gastroenterol..

[38] Dong H.-C., Cui X.-B., Wang L.-H., Li M., Shen Y.-Y., Zhu J.-B., Li C.-F., Hu J.-M., Li S.-G., Yang L. (2015). Type-specific detection of human papillomaviruses in Kazakh esophageal squamous cell carcinoma by genotyping both E6 and L1 genes with MALDI-TOF mass spectrometry. Int. J. Clin. Exp. Pathol..

[39] Farhadi M., Tahmasebi Z., Merat S., Kamangar F., Nasrollahzadeh D., Malekzadeh R. (2005). Human papillomavirus in squamous cell carcinoma of esophagus in a high-risk population. World J. Gastroenterol..

[40] Georgantis G., Syrakos T., Agorastos T., Miliaras S., Gagalis A., Tsoulfas G., Spanos K., Marakis G. (2015). Detection of human papillomavirus DNA in esophageal carcinoma in Greece. World J. Gastroenterol..

[41] Gessner A.L., Borkowetz A., Baier M., Göhlert A., Wilhelm T.J., Thumbs A., Borgstein E., Jansen L., Beer K., Mothes H. (2018). Detection of HPV16 in esophageal cancer in a high-incidence region of Malawi. Int. J. Mol. Sci..

[42] Han C., Qiao G., Hubbert N.L., Li L., Sun C., Wang Y., Yan M., Xu D., Li Y., Lowy D.R. (1996). Serologic association between human papillomavirus type 16 infection and esophageal cancer in Shaanxi Province, China. J. Natl. Cancer Inst..

[43] He Z., Xu Z., Hang D., Guo F., Abliz A., Weiss N.S., Xi L., Liu F., Ning T., Pan Y. (2014). Anti-HPV-E7 seropositivity and risk of esophageal squamous cell carcinoma in a high-risk population in China. Carcinogenesis.

[44] Jiang H.Y., Feng D.Y., Yan Y.H., Zheng H. (2005). The Relation of Human Papillomavirus (HPV) and Esophageal Squamous Cell Carcinogenesis. J. Clin. Res..

[45] Kamangar F., Qiao Y.-L., Schiller J.T., Dawsey S.M., Fears T., Sun X.-D., Abnet C.C., Zhao P., Taylor P.R., Mark S.D. (2006). Human papillomavirus serology and the risk of esophageal and gastric cancers: Results from a cohort in a high-risk region in China. Int. J. Cancer.

[46] Kawaguchi H., Ohno S., Araki K., Miyazaki M., Saeki H., Watanabe M., Tanaka S., Sugimachi K. (2000). p53 polymorphism in human papillomavirus-associated esophageal cancer. Cancer Res..

[47] Kayamba V., Bateman A.C., Asombang A.W., Shibemba A., Zyambo K., Banda T., Soko R., Kelly P. (2015). HIV infection and domestic smoke exposure, but not human papillomavirus, are risk factors for esophageal squamous cell carcinoma in Zambia: A case-control study. Cancer Med..

[48] Khurshid A., Kazuya N., Hanae I., Manabu I. (1998). Infection of human papillomavirus (HPV) and Epstein-Barr virus (EBV) and p53 expression in human EC. J. Pak. Med. Assoc..

[49] Kuang Z.S., Tang C.Z., Shen Z.Y. (2000). HPV16, 18 detection in esophageal cancer. Guangdong Med..

[50] Lagergren J., Wang Z., Bergström R., Dillner J., Nyrén O. (1999). Human papillomavirus infection and esophageal cancer: A nationwide seroepidemiologic case-control study in Sweden. J. Natl. Cancer Inst..

[51] Li Y., Huang G.Q., Xiao H.Y., Hong Y.F., Mao T., Deng W.J. (1991). Esophageal tissue adjacent to carcinoma and the presence of human papilloma virus DNA. Huaxi Med. Univ. J..

[52] Liu H.Y., Zhou S.L., Ku J.W., Zhang D.Y., Li B., Na Han X., Fan Z.M., Cui J.L., Lin H.L., Guo E.T. (2014). Prevalence of human papillomavirus infection in esophageal and cervical cancers in the high incidence area for the two diseases from 2007 to 2009 in Linzhou of Henan Province, Northern China. Arch. Virol..

[53] Liu J., Su Q., Zhang W. (2000). Relationship between HPV-E6 P53 protein and esophageal squamous cell carcinoma. World J. Gastroenterol..

[54] Liyanage S.S., Segelov E., Malik A., Garland S.M., Tabrizi S.N., Cummins E., Seale H., Rahman B., Moa A., Barbour A.P. (2014). A case-control study of the role of human papillomavirus in oesophageal squamous cell carcinoma in Australia. J. Oncol..

[55] Lu L.C., Shen Z.Y., You S.J., Shen J., Cai W.Y. (1999). Detection of human papillomavirus (HPV) on EC by PCR and in-situ hybridization. Chin. J. Cancer.

[56] Lu X.M., Zhang Y.M., Lin R.Y., Liang X.H., Zhang Y.L., Wang X., Zhang Y., Wang Y., Wen H. (2004). p53 polymorphism in human papillomavirus-associated Kazakh’s esophageal cancer in Xinjiang, China. World J. Gastroenterol..

[57] Lyronis I.D., Baritaki S., Bizakis I., Krambovitis E., Spandidos D.A. (2008). K-ras mutation, HPV infection and smoking or alcohol abuse positively correlate with esophageal squamous carcinoma. Pathol. Oncol. Res..

[58] Parameshwaran K., Sharma P., Rajendra S., Stelzer-Braid S., Xuan W., Rawlinson W.D. (2019). Circulating human papillomavirus DNA detection in Barrett’s dysplasia and esophageal adenocarcinoma. Dis. Esophagus..

[59] Rajendra S., Xuan W., Hufnagel K., Sharma P., Pavey D., Alhajjiri N., Rattan A., Wang B. (2020). Antibodies against human papillomavirus proteins in Barrett’s dysplasia and intramucosal esophageal adenocarcinoma. Ann. N. Y. Acad. Sci..

[60] Ren Z.H.P., Shi Z., Chen W.L. (1996). HPV16, 18E6 protein and p21 ras p53 expression in esophageal cancer tissues. Chin. J. Clin. Exp. Pathol..

[61] Sadeghian Z., Baghi H.B., Poortahmasebi V., Sadeghi J., Hasani A., Azadi A., Oskouee M.A. (2022). Evidence of High-Risk Human Papillomavirus in Esophageal Cancer in East Azerbaijan Province, Northwest of Iran. Can J. Infect. Dis. Med. Microbiol..

[62] Shen Z.-Y., Xu L.-Y., Li E.-M., Cai W.-J., Shen J., Chen M.-H., Cen S., Tsao S.-W., Zeng Y. (2004). The multistage process of carcinogenesis in human esophageal epithelial cells induced by human papillomavirus. Oncol. Rep..

[63] Tornesello M.L., Monaco R., Nappi O., Buonaguro L., Buonaguro F.M. (2009). Detection of mucosal and cutaneous human papillomaviruses in oesophagitis, squamous cell carcinoma and adenocarcinoma of the oesophagus. J. Clin. Virol..

[64] Wang X.J., Wang C.J., Wang X.H., Tao D.M., Xiao H.Z. (1996). Etiologic Relationship between Human Papillomavirus and Esophageal Cancer. Chin. J. Clin. Oncol..

[65] Wong M.Y.W., Wang B., Yang A., Khor A., Xuan W., Rajendra S. (2018). Human papillomavirus exposure and sexual behavior are significant risk factors for Barrett’s dysplasia/esophageal adenocarcinoma. Dis. Esophagus.

[66] Xu C.-L., Qian X.-L., Zhou X.-S., Zhao Q.-Z., Li Y.-C. (2004). Expression of HPV 16-E6 and E7 oncoproteins in squamous cell carcinoma tissues of esophageal cancer and non-cancer tissues. Chin. J. Cancer.

[67] Xu W.-G., Zhang L.-J., Lu Z.-M., Li J.-Y., Ke Y., Xu G.-W. (2003). Detection of human papillomavirus type 16 E6 mRNA in carcinomas of upper digestive tract. Zhonghua Yi Xue Za Zhi.

[68] Yahyapour Y., Rahmani R., Alipour M., Alizadeh A., Khademian A., Sadeghi F. (2018). Prevalence and association of human papillomavirus, Epstein-Barr virus and Merkel Cell polyomavirus with neoplastic esophageal lesions in northern Iran. Casp. J. Intern Med..

[69] Yang J., Wu H., Wei S., Xiong H., Fu X., Qi Z., Jiang Q., Li W., Hu G., Yuan X. (2014). HPV seropositivity joints with susceptibility loci identified in GWASs at apoptosis associated genes to increase the risk of Esophageal Squamous Cell Carcinoma (ESCC). BMC Cancer.

[70] Zhou S.-M., Sheyhidin I., Yang T., Zhang L.-W., Hasim A., Lu X.-M., Niyaz M., Liu T. (2009). Relationship between human papillomavirus 16 and esophageal squamous cell carcinoma in Uygur population in Xinjiang Uygur Autonomous Region. World Chin. J. Dig..

[71] Zhou X.-B., Guo M., Quan L.-P., Zhang W., Lu Z.-M., Wang Q.-H., Ke Y., Xu N.Z. (2003). Detection of human papillomavirus in Chinese esophageal squamous cell carcinoma and its adjacent normal epithelium. World J. Gastroenterol..

[72] Ludmir E.B., Stephens S.J., Palta M., Willett C.G., Czito B.G. (2015). Human papillomavirus tumor infection in esophageal squamous cell carcinoma. J. Gastrointest. Oncol..

[73] Wang J., Zhao L., Yan H., Che J., Huihui L., Jun W., Liu B., Cao B. (2016). A meta-analysis and systematic review on the association between human papillomavirus (types 16 and 18) infection and esophageal cancer worldwide. PLoS ONE.

[74] International Agency for Research on Cancer Global Cancer Observatory, Estimated age-standardized incidence and mortality rates (World) in 2020. http://globocan.iarc.fr/Pages/fact_sheets_cancer.aspx?cancer=stomach.

[75] Jemal A., Bray F., Center M.M., Ferlay J., Ward E., Forman D. (2011). Global cancer statistics. CA Cancer J. Clin..

[76] Snietura M., Waniczek D., Piglowski W., Kopec A., Nowakowska-Zajdel E., Lorenc Z., Muc-Wierzgon M. (2014). Potential role of human papilloma virus in the pathogenesis of gastric cancer. World J. Gastroenterol..

[77] Zeng Z.-M., Luo F.-F., Zou L.-X., He R.-Q., Pan D.-H., Chen X., Xie T.-T., Li Y.-Q., Peng Z.-G., Chen G. (2016). Human papillomavirus as a potential risk factor for gastric cancer: A meta-analysis of 1,917 cases. OncoTargets Ther..

[78] Wang H., Chen X.-L., Liu K., Bai D., Zhang W.-H., Chen X.-Z., Hu J.-K., SIGES Research Group (2020). Associations Between Gastric Cancer Risk and Virus Infection Other Than Epstein-Barr Virus: A Systematic Review and Meta-analysis Based on Epidemiological Studies. Clin. Transl. Gastroenterol..

[79] WHO Global Cancer Observatory. https://gco.iarc.fr/today/home.

[80] Johnson C.M., Wei C., Ensor J.E., Smolenski D.J., Amos C.I., Levin B., Berry D.A. (2013). Meta-analyses of colorectal cancer risk factors. Cancer Causes Control.

[81] Aghakhani A., Hamkar R., Ramezani A., Bidari-Zerehpoosh F., Sabeti S., Ghavami N., Banifazl M., Rashidi N., Eslamifar A. (2014). Lack of human papillomavirus DNA in colon adenocarcinama and adenoma. J. Cancer Res. Ther..

[82] Audeau A., Han H.W., Johnston M.J., Whitehead M.W., Frizelle F.A. (2002). Does human papilloma virus have a role in squamous cell carcinoma of the colon and upper rectum?. Eur. J. Surg. Oncol..

[83] Bernabe-Dones R.D., Gonzalez-Pons M., Villar-Prados A., Lacourt-Ventura M., Rodríguez-Arroyo H., Fonseca-Williams S., Velazquez F.E., Diaz-Algorri Y., Lopez-Diaz S.M., Rodríguez N. (2016). High Prevalence of Human Papillomavirus in Colorectal Cancer in Hispanics: A Case-Control Study. Gastroenterol. Res. Pract..

[84] Bodaghi S., Yamanegi K., Xiao S.Y., Da Costa M., Palefsky J.M., Zheng Z.M. (2005). Colorectal papillomavirus infection in patients with colorectal cancer. Clin. Cancer Res..

[85] Boguszaková L., Hirsch I., Brichácek B., Faltýn J., Fric P., Dvoráková H., Vonka V. (1988). Absence of cytomegalovirus, Epstein-Barr virus, and papillomavirus DNA from adenoma and adenocarcinoma of the colon. Acta Virol..

[86] Buyru N., Tezol A., Dalay N. (2006). Coexistence of K-ras mutations and HPV infection in colon cancer. BMC Cancer.

[87] Cheng J.Y., Sheu L.F., Meng C.L., Lee W.H., Lin J.C. (1995). Detection of human papillomavirus DNA in colorectal carcinomas by polymerase chain reaction. Gut.

[88] Dalla Libera L.S., de Siqueira T., Santos I.L., Porto Ramos J.E., Milhomen A.X., Alencar R.C.G., Rabelo Santos S.H., Dos Santos Corneiro M.A., Figueiredo Alves R.R., Saddi V.A. (2020). Detection of Human papillomavirus and the role of p16INK4a in colorectal carcinomas. PLoS ONE.

[89] Gazzaz F., Mosli M.H., Jawa H., Sibiany A. (2016). Detection of human papillomavirus infection by molecular tests and its relation to colonic polyps and colorectal cancer. Saudi Med. J..

[90] Gornick M.C., Castellsague X., Sanchez G.I., Giordano T.J., Vinco M., Greenson J.K., Capella G., Raskin L., Rennert G., Gruber S.B. (2010). Human papillomavirus is not associated with colorectal cancer in a large international study. Cancer Causes Control.

[91] Hafez F.S., Meckawy G.R., Alorabi M., Shakweer M.M. (2022). Interpretation of P16 expression as a marker of HPV in colorectal cancer. Histol. Histopathol..

[92] Kirgan D., Manalo P., Hall M., McGregor B. (1990). Association of human papillomavirus and colon neoplasms. Arch Surg..

[93] Laskar R.S., Talukdar F.R., Choudhury J.H., Singh S.A., Kundu S., Dhar B., Mondal R., Ghosh S.K. (2015). Association of HPV with genetic and epigenetic alterations in colorectal adenocarcinoma from Indian population. Tumor Biol..

[94] Lee Y.M., Leu S.Y., Chiang H., Fung C.P., Liu W.T. (2001). Human papillomavirus type 18 in colorectal cancer. J. Microbiol. Immunol. Infect..

[95] Li Y.X., Zhang L., Simayi D., Zhang N., Tao L., Yang L., Zhao J., Chen Y.Z., Li F., Zhang W.J. (2015). Human papillomavirus infection correlates with inflammatory Stat3 signaling activity and IL-17 level in patients with colorectal cancer. PLoS ONE.

[96] Liu F., Mou X., Zhao N., Lin J., Teng L., Xiang C. (2011). Prevalence of human papillomavirus in Chinese patients with colorectal cancer. Color. Dis..

[97] Mahmoudvand S., Safaei A., Erfani N., Sarvari J. (2015). Presence of Human Papillomavirus DNA in Colorectal Cancer Tissues in Shiraz, Southwest Iran. Asian Pac. J. Cancer Prev..

[98] McGregor B., Byrne P., Kirgan D., Albright J., Manalo P., Hall M. (1993). Confirmation of the association of human papillomavirus with human colon cancer. Am. J Surg..

[99] Militello V., Trevisan M., Squarzon L., Biasolo M.A., Rugge M., Militello C., Palù G., Barzon L. (2009). Investigation on the presence of polyomavirus, herpesvirus, and papillomavirus sequences in colorectal neoplasms and their association with cancer. Int. J. Cancer.

[100] Nagi K., Gupta I., Jurdi N., Yasmeen A., Vranic S., Batist G., Moustafa A.-E. (2021). Copresence of high-risk human papillomaviruses and Epstein-Barr virus in colorectal cancer: A tissue microarray and molecular study from Lebanon. Int. J. Mol. Sci..

[101] Pérez L.O., Abba M.C., Laguens R.M., Golijow C.D. (2005). Analysis of adenocarcinoma of the colon and rectum: Detection of human papillomavirus (HPV) DNA by polymerase chain reaction. Color. Dis..

[102] Picanço-Junior O.M., Oliveira A.L., Freire L.T., Brito R.B., Villa L.L., Matos D. (2014). Association between human Papillomavirus and colorectal adenocarcinoma and its influence on tumor staging and degree of cell differentiation. Arq. Bras. Cir. Dig..

[103] Ranjbar R., Saberfar E., Shamsaie A., Ghasemian E. (2014). The aetiological role of human papillomavirus in colorectal carcinoma: An Iranian population-based case control study. Asian Pac. J. Cancer Prev..

[104] Salepci T., Yazici H., Dane F., Topuz E., Dalay N., Onat H., Aykan F., Seker M., Aydiner A. (2009). Detection of human papillomavirus DNA by polymerase chain reaction and southern blot hybridization in colorectal cancer patients. J. BUON.

[105] Snietura M., Waniczek D., Nowakowska-Zajdel E., Piglowski W., Kopec A., Muc-Wierzgon M. (2012). Does human papilloma virus participate in colorectal carcinogenesis?. J. Biol. Regul. Homeost. Agents.

[106] Soto Y., Limia C.M., González L., Grá B., Hano O.M., Martínez P.A., Kourí V. (2016). Molecular evidence of high-risk human papillomavirus infection in colorectal tumours from Cuban patients. Mem. Inst. Oswaldo Cruz..

[107] Taherian H., Tafvizi F., Fard Z.T., Abdirad A. (2014). Lack of association between human papillomavirus infection and colorectal cancer. Prz. Gastroenterol..

[108] Tanzi E., Bianchi S., Frati E.R., Amicizia D., Martinelli M., Bragazzi N.L., Brisigotti M.P., Colzani D., Fasoli E., Zehender G. (2015). Human papillomavirus detection in paraffin-embedded colorectal cancer tissues. J. Gen. Virol..

[109] Vuitton L., Jaillet C., Jacquin E., Monnien F., Heberle M., Mihai M.I., Lassabe C., Raffoul J., Puyraveau M., Lakkis Z. (2017). Human papillomaviruses in colorectal cancers: A case-control study in western patients. Dig. Liver Dis..

[110] Zhang J., Ding Y., Zhou Z., Li H., Zhou B. (2005). Expression of human papillomavirus 16 E7 DNA in patients with colorectal adenocarcinoma. Sheng Wu Yi Xue Gong Cheng Xue Za Zhi.

[111] Zhu Q., Cao J., Li S. (1999). Detection of human papillomavirus gene in biopsies from colon carcinoma by PCR. Zhonghua Shi Yan He Lin Chuang Bing Du Xue Za Zhi.

[112] Hill A.B. (1965). The environment and disease: Association or causation?. Proc. R Soc. Med..

[113] Baandrup L., Thomsen L.T., Olesen T.B., Andersen K.K., Norrild B., Kjaer S.K. (2014). The prevalence of human papillomavirus in colorectal adenomas and adenocarcinomas: A systematic review and meta-analysis. Eur. J. Cancer.

[114] Damin D.C., Ziegelmann P.K., Damin A.P. (2013). Human papillomavirus infection and colorectal cancer risk: A meta-analysis. Color. Dis..

[115] Wieland U., Kreuter A. (2019). Anal cancer risk: HPV-based cervical screening programmes. Lancet Infect. Dis..

[116] Krzowska-Firych J., Lucas G., Lucas C., Lucas N., Pietrzyk Ł. (2019). An overview of Human Papillomavirus (HPV) as an etiological factor of the anal cancer. Infect. Public Health.

[117] Joseph D.A., Miller J.W., Wu X., Chen V.W., Morris C.R., Goodman M.T., Villalon-Gomez J.M., Williams M.A., Cress R.D. (2008). Understanding the burden of human papillomavirus-associated anal cancers in the US. Cancer.

[118] Gomez D.T., Santos J.L. (2007). Human papillomavirus infection and cervical cancer: Pathogenesis and epidemiology. Appl. Microbiol..

[119] Beachler D.C., Kreimer A.R., Schiffman M., Herrero R., Wacholder S., Rodriguez A.C., Lowy D.R., Porras C., Schiller J.T., Quint W. (2015). Costa Rica HPV Vaccine Trial (CVT) Group. Multisite HPV16/18 vaccine efficacy against cervical, anal, and oral HPV infection. J. Natl. Cancer Inst..

